# Biomimetic Nanomaterials: Diversity, Technology, and Biomedical Applications

**DOI:** 10.3390/nano12142485

**Published:** 2022-07-20

**Authors:** Kamil G. Gareev, Denis S. Grouzdev, Veronika V. Koziaeva, Nikita O. Sitkov, Huile Gao, Tatiana M. Zimina, Maxim Shevtsov

**Affiliations:** 1 Department of Micro and Nanoelectronics, Saint Petersburg Electrotechnical University “LETI”, 197022 Saint Petersburg, Russia; sitkov93@yandex.ru (N.O.S.); tmzimina@gmail.com (T.M.Z.); 2 Laboratory of Biomedical Nanotechnologies, Institute of Cytology of the Russian Academy of Sciences, 194064 Saint Petersburg, Russia; 3 SciBear OU, Tartu mnt 67/1-13b, Kesklinna Linnaosa, 10115 Tallinn, Estonia; denisgrouzdev@gmail.com; 4 Research Center of Biotechnology of the Russian Academy of Sciences, Institute of Bioengineering, 119071 Moscow, Russia; vkoziaieva@mail.ru; 5 Key Laboratory of Drug-Targeting and Drug Delivery System of the Education Ministry, West China School of Pharmacy, Sichuan University, Chengdu 610041, China; gaohuile@scu.edu.cn; 6 Center of Translational Cancer Research (TranslaTUM), Klinikum Rechts der Isar, Technical University Munich, 81675 Munich, Germany; 7 Personalized Medicine Centre, Almazov National Medical Research Centre, 197341 Saint Petersburg, Russia; 8 National Center for Neurosurgery, Nur-Sultan 010000, Kazakhstan

**Keywords:** biomimetics, nanomaterials, nanoparticles, synthesis technique, applications, biomedicine, theranostics

## Abstract

Biomimetic nanomaterials (BNMs) are functional materials containing nanoscale components and having structural and technological similarities to natural (biogenic) prototypes. Despite the fact that biomimetic approaches in materials technology have been used since the second half of the 20th century, BNMs are still at the forefront of materials science. This review considered a general classification of such nanomaterials according to the characteristic features of natural analogues that are reproduced in the preparation of BNMs, including biomimetic structure, biomimetic synthesis, and the inclusion of biogenic components. BNMs containing magnetic, metal, or metal oxide organic and ceramic structural elements (including their various combinations) were considered separately. The BNMs under consideration were analyzed according to the declared areas of application, which included tooth and bone reconstruction, magnetic and infrared hyperthermia, chemo- and immunotherapy, the development of new drugs for targeted therapy, antibacterial and anti-inflammatory therapy, and bioimaging. In conclusion, the authors’ point of view is given about the prospects for the development of this scientific area associated with the use of native, genetically modified, or completely artificial phospholipid membranes, which allow combining the physicochemical and biological properties of biogenic prototypes with high biocompatibility, economic availability, and scalability of fully synthetic nanomaterials.

## 1. Introduction

“Biomimetics” is a concept introduced in 1957 by Otto Schmitt [1,2] that, generally, means the imitation of structures, characteristics, models, and compositions of natural objects to solve various problems [3]. The word “biomimetic” comes from the Greek word “bios” (life) and the suffix “mimetic” (mimicry) [4]. This concept also includes approaches and processes mimicking biological or natural ones such as, for example, the synthesis of nanomaterials or nanostructures that reproduce the physicochemical, mechanical, and biological properties of natural (biogenic) materials at the nano- or macroscale [5,6]. Similar to the formation of biogenic materials, the technology of biomimetic materials includes the processes of self-assembly and interfacial molecular recognition [7,8]. Other important directions of development for such materials are biopolymers [9] and biocomposites [10], which are receiving immense consideration in biomedical and other fields.

**Figure 1:**
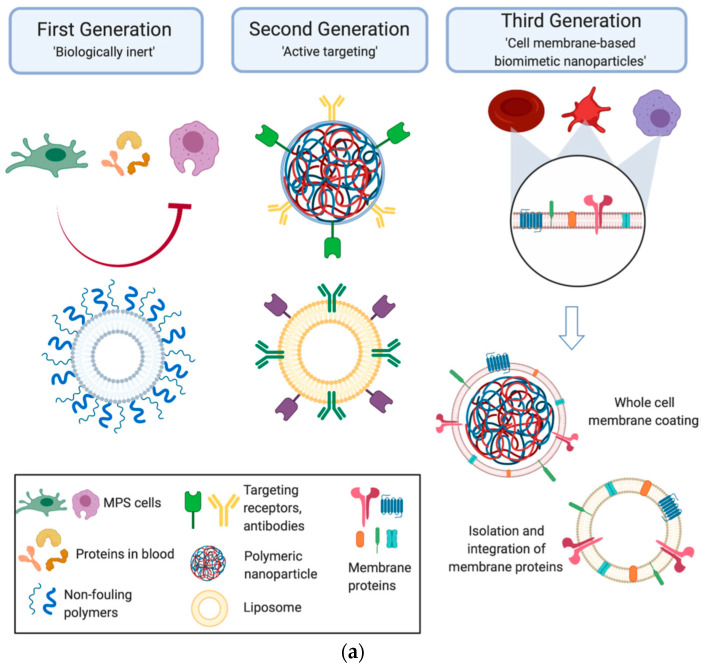

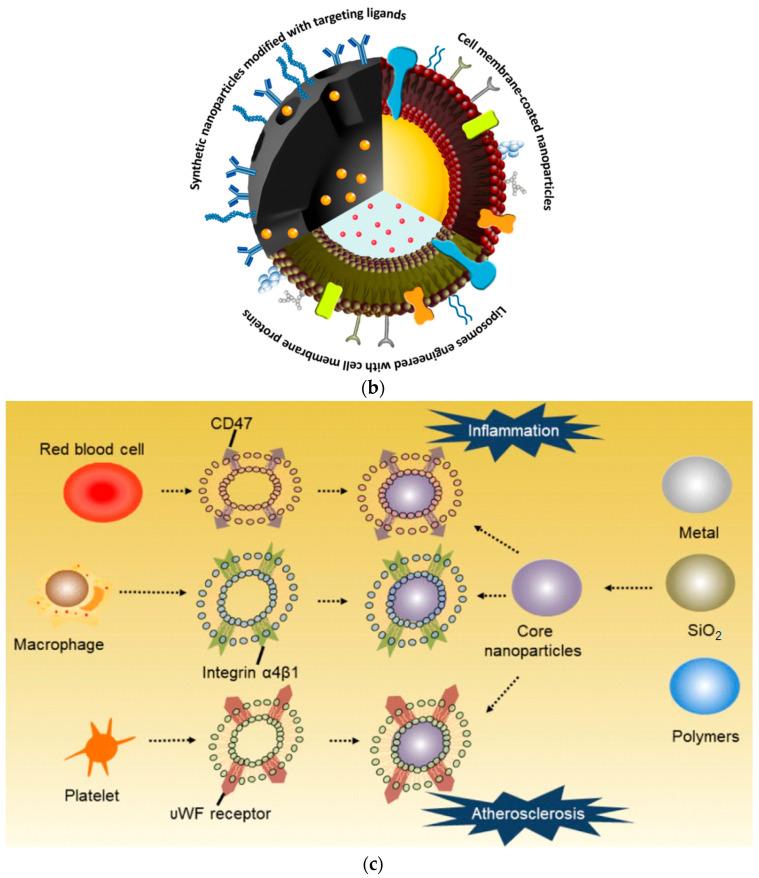
Biomimetic nanoparticles for biomedical applications. (**a**) Technology evolution: Early generations of particles were biologically inert and covered with nonfouling coatings, preventing their nonspecific interactions with the cells they encountered *in vivo*. From here, the next generation of nanoparticles became active, targeting molecules, which enabled the nanoparticles to reach the disease site and engage with the local environment. Taking inspiration from nature, the third generation of cell-membrane-based biomimetic nanoparticles mimicked the surface features of native cells by utilizing the whole cell membrane or membrane protein functionalization onto synthetic carriers (Reprinted from [31], license CC BY 4.0.) (**b**) Schematic presentation of different strategies of inflammation-targeting biomimetic nanoparticles. Orange and red spheres represent drug-encapsulated synthetic nanoparticles (grey) and liposomes (green), respectively. (Reprinted from [16], license CC BY 4.0.) (**c**) An example of modern BNM concept implementation: cell-membrane-coated NPs designed for atherosclerosis and inflammation therapy. The membranes of RBCs, platelets, and macrophages are extracted and used to coat different kinds of NPs depending on the site of inflammation and atherosclerosis. Each cell membrane has its own unique surface proteins, such as CD47 on RBC, integrin a4b1 on macrophages, and GPIIb/IIa on platelets, modifying the therapeutic effects. (Reprinted from [20], license CC BY 4.0.)

At the same time, the process of biomimetic material formation can differ greatly from the natural analog process in terms of parameters (for example, high energies during plasma deposition [11] or electric currents during electrospinning [12]). The transfer of an idea or mechanism from living systems to nonliving ones is not trivial. The direct copying of a biological prototype may not be successful, even if it is feasible with modern technology [13]. Currently, biomimetic materials play an important role in medical science in the development of drug delivery systems and theranostics [14,15,16,17,18,19,20,21,22,23,24,25,26], tissue engineering [27,28,29], and combined solutions to these problems [30]. Chronologically, the first attempts of delivery-system-mimicking were directed towards the development of “biologically inert” nanoparticles (NPs), demonstrating reduced interaction with immune cells. The next generation of delivery system had to provide immune inertness and specific binding to target pathogenic cells, such as cells present in pathological lesions in neurodegenerative disorders, inflammatory endothelial cells, etc. These tasks required the development of “targeted” NPs functionalized with monoclonal antibodies or other types of ligands. The next step was the development of biomimetic NPs partially complementary to target cells that reproduced the surface characteristics of native cells, which further improved the effectiveness of delivery systems [31]. A schematic diagram illustrating the evolution of NP technology for biomedicine is shown in Figure 1a.

An example of the technological evolution of NPs is the synthesis of biomimetic nanomaterials (BNMs) that specifically accumulate in the inflammatory zone. Over the past thirty years, the development of such NPs has largely relied on three strategies [16]:

(1) Synthetic NPs modified with targeting ligands that mimic cell surface proteins;
(2) NPs covered with a native cell membrane;
(3) Liposomes formed using cell membrane proteins (Figure 1b).

Biomimetic NPs take advantage of both cell membranes and synthetic NPs and are distinguished by three main features: prolonged circulation in the bloodstream, specific binding, and reduced toxicity [16]. BNMs intended for the targeted therapy of atherosclerosis and inflammatory diseases can be coated with cell membranes derived from various types of cells, such as erythrocytes, macrophages, and platelets. The methods for obtaining cell-membrane-coated NPs can be summarized as a three-step process. At the first stage, the cell membrane is isolated from the original cells (cells can be lysed using various methods, after which centrifugation is used to separate fragments of the cell membrane). Then, the prepared cell wall fragments are repeatedly pressed through a polycarbonate membrane with a pore diameter of ~200–400 nm to obtain microcapsules from the cell membrane, and an NP core with a dissolved drug is also prepared. In the last step, NPs are encapsulated into microcapsules made of the cell membrane (see Figure 1c) [20].

**Figure 2:**
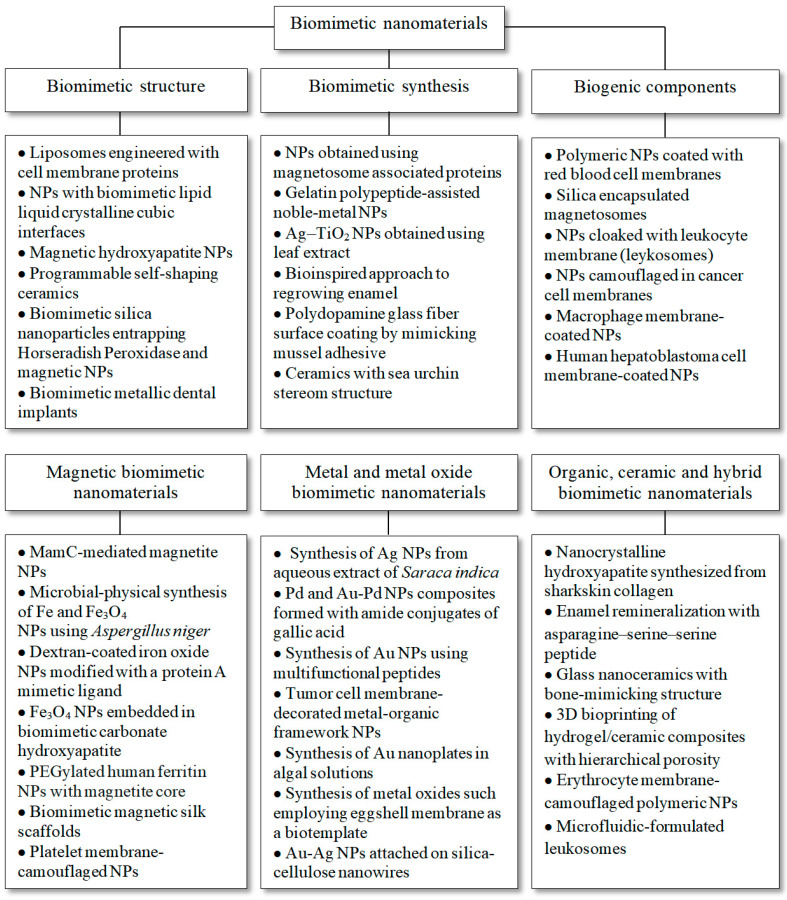
Classification of biomimetic nanomaterials (BNMs) based on the literature data, including information on biomimetic structure [16,34,35,36,37,38], biomimetic synthesis [32,39,40,41,42,43], biogenic components [31,33,44,45,46,47], magnetic BNMs [48,49,50,51,52,53,54], metal and metal oxide BNMs [55,56,57,58,59,60,61], organic, ceramic and hybrid BNMs [62,63,64,65,66,67].

Based on the above, we propose a general classification of BNMs based on the purposes of reproduced BNMs and defined as materials with biomimetic structural elements (for example, liposomes incorporating cell wall proteins [16]), materials produced using biomimetic methods (for example, NPs grown using magnetosome-associated proteins [32]), and materials containing biogenic components, i.e., so-called nanobiohydrides [9] (e.g., polymeric NPs coated with erythrocyte cell membranes [33]). Due to the wide variety of proposed structures and compositions of BNMs, the use of magnetic, metal, and metal oxide materials, as well as organic, ceramic, and hybrid (multicomponent) structural elements, are considered separately. The proposed classification with some examples from the literature, including information on biomimetic structure [16,34,35,36,37,38], biomimetic synthesis [32,39,40,41,42,43], biogenic components [31,33,44,45,46,47], magnetic BNMs [48,49,50,51,52,53,54], metal and metal oxide BNMs [55,56,57,58,59,60,61], organic, ceramic and hybrid BNMs [62,63,64,65,66,67], is shown in Figure 2.

Thus, in this review, BNMs are understood as functional materials containing nanosized components and having structural and technological similarities to natural (biogenic) analogues.

## 2. Interaction between Biomimetic Nanomaterials and Biological Tissue

**Figure 3:**
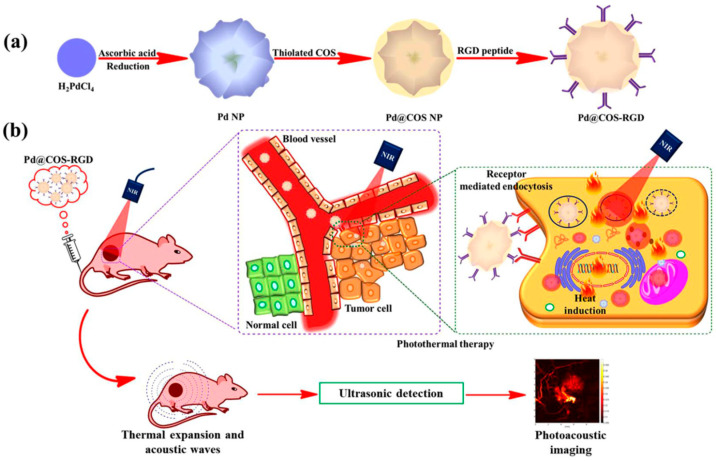
Chitosan-oligosaccharide-coated biocompatible palladium nanoparticles (Pd@COS NPs) for photo-based imaging and therapy. (**a**) A scheme showing the preparation of Pd NPs, further surface coating with thiolated chitosan oligosaccharide (Pd@COS NPs), and finally, functionalization using an RGD peptide (Pd@COS-RGD). (**b**) A systematic illustration showing the photothermal ablation and photoacoustic imaging of tumor tissue using Pd@COS-RGD. (Reprinted from [69], license CC BY 4.0.)

The shapes, sizes, and surface characteristics of BNMs provide outstanding advantages that enable them to deliver water-insoluble drugs, prevent premature drug release *in vivo*, enhance biodistribution, control drug release, improve pharmacokinetics, and increase intracellular penetration. Due to their small sizes, these particles are able to pass through the finest blood capillaries to enable passive targeting of tumors, as enhanced permeability and retention can affect tumor vasculature [68]. Untargeted theranostic agents reduce therapeutic efficiency by nonspecific accumulation in other tissues. There are two possible mechanisms to improve the tumor selectivity of nanoparticles: (i) passive targeting through enhanced permeability retention and (ii) active targeting based on a specific cell-surface receptor with a ligand on the nanoparticles. Integrins, heterodimeric cell adhesion proteins involved in many mechanisms, including cell attachment, angiogenesis, and the metastasis of solid tumors, have been identified for the active targeting of tumor tissue in clinical trials. The authors of [69] synthesized chitosan-oligosaccharide-coated biocompatible palladium nanoparticles for photo-based imaging and therapy with effective accumulation in breast cancer cells. Further, the NIR-based photothermal ablation and PAT imaging efficiency of the formulated particles were examined using *in vitro* and *in vivo* models, as shown in Figure 3.

Conventional silica NPs are widely applied in drug delivery systems and as optical contrast agents for imaging due to their water solubility, chemical and thermal stability, low toxicity, and good biocompatibility; however, the functionalization of silica NPs is often limited by their surface properties. Biotemplated mesoporous silica NPs exhibit a higher surface area and a tunable pore volume, allowing for a higher therapeutic drug-loading capacity. A variety of methods have been established to prepare mesoporous silica NPs. Although the silica wall is amorphous, the interior of the material possesses an extremely ordered framework with uniform mesopores. It was proposed that cationic surfactant molecules could self-organize into a hexagonal structure to serve as a template, followed by the co-condensation of silica precursors and cylindrical micelles to form mesoporous silica nanoparticles (MSNs) with a pore size range of 2–50 nm [70]. Based on recent developments in nanomaterials research, silicon-based nanomaterials, along with combinations of silicon-based nanomaterials and other nanomaterials, have gained attention also in noninvasive disease diagnosis. MSNs can be suitable platforms for imaging agents because of their highly specific surface areas and functionalized surfaces [22]. Yang et al. [71] proposed a more complex schematic illustration of the composition and architecture of engineered silica-based nanoparticles with diverse appealing properties as nanocarriers, including cellular uptake, DNA transfection, adhesion and transcytosis, and biomodulators, including dendritic cell maturation, cancer cell immunogenicity, macrophage polarization, and tumor microenvironment, for biomedical applications. Cage-like polyhedral oligomeric silsesquioxanes, owing to their biocompatibility and ability to incorporate with different polymers, were shown to offer high potential for several biomedical applications such as drug delivery, dental composites, biosensors, biomedical devices, and tissue engineering [72].

It was shown in [73] that organic–inorganic hybrid polydopamine-chitosan-coated AgNPs obtained via an mussel-inspired electropolymerization strategy could be used for the noninvasive electrochemical detecting of a malondialdehyde biomarker. This result confirmed the perspectives of BNM uses both *in vivo* and *in vitro*. Another approach for understanding BNM and tissue interaction was proposed in [74], where the authors considered some typical examples of human bovine serum albumin, ferritin, and human transferrin-based nanoplatforms loaded with noble metals, oxides, semiconductors, and polymers for multifunctional applications, including chemodynamic therapy, chemotherapy, X-ray computed tomography, fluorescent imaging, magnetic resonance imaging, photoacoustic therapy, photodynamic therapy, positron emission computed tomography, photothermal therapy, and radiotherapy.

**Figure 4:**
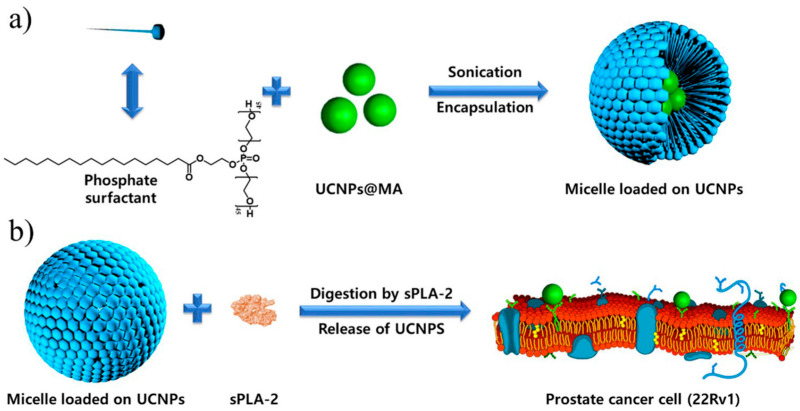
Schematic illustration of encapsulation procedure and release mechanism of biocompatible upconversion nanoparticles (UCNP). (**a**) Encapsulation of UCNPs in a novel, synthesized phosphate surfactant through sonication at rt. (**b**) Release of UCNPs after a specific cleavage of phosphate surfactant by the sPLA-2 enzyme. (Reprinted from [75], license CC BY 4.0.)

Imaging methods using upconversion nanoparticles (UCNPs) have been proposed as prospective diagnosis tools due to their ability to absorb near-infrared radiation and transform it into visible light through an upconversion process of multiple-photon absorption. Sharipov et al. [75] developed novel UCNP-loaded phosphate micelles that could be cleaved by the secreted phospholipase A2 (sPLA-2) enzyme, allowing the release and delivery of UCNPs directly to prostate cancer cells. The ability to release UCNPs in a precise location provided several advantages, including the efficient delivery of UCNPs in low concentrations, an increase in dispersion, and a remarkably high selectivity to prostate cancer cells. Correspondingly, these benefits could reduce biological side effects that other delivery systems have faced. Nanoparticles were prepared with a hydrothermal method, and a delivery system was developed in the form of phosphate micelles synthesized from biocompatible materials (see Figure 4).

**Figure 5:**
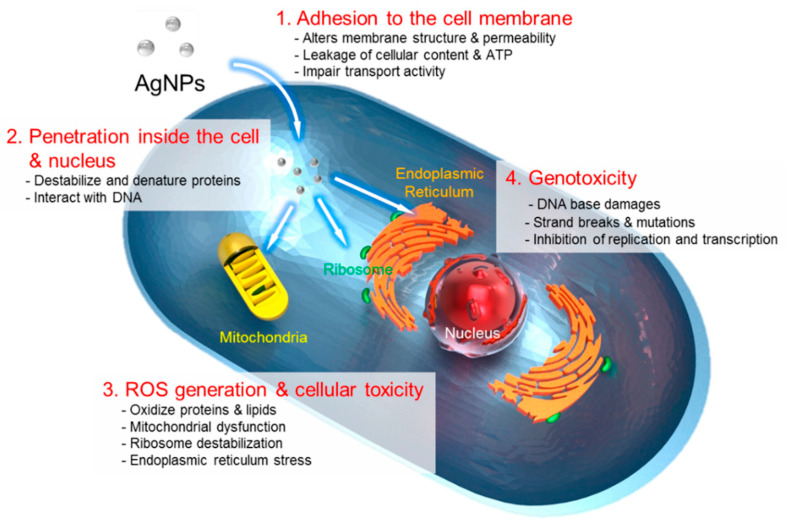
The four main routes of the cytotoxic mechanism of AgNPs. 1: AgNPs adhere to the surface of a cell, damaging its membrane and altering the transport activity; 2: AgNPs and Ag ions penetrate inside the cell and interact with numerous cellular organelles and biomolecules, which can affect corresponding cellular functions; 3: AgNPs and Ag ions participate in the generation of reactive oxygen species (ROS) inside the cell, leading to cell damage; and 4: AgNPs and Ag ions induce the genotoxicity. (Reprinted from [81], license CC BY 4.0.)

Renu et al. [76] considered green-chemistry (plant extract)-mediated synthesized metal nanoparticles (silver nanoparticles) and incorporated biodegradable polymeric nanocomposite preparation, characterization, and its mechanism of antimicrobial-based wound-healing activity. Their examination of the literature displayed that there are two key types of nanocomposites of metals with polymers: (1) inorganic, metal-core nanoparticles enclosed in a polymer shell and (2) inorganic metal nanoparticles inserted into a polymer matrix. Transition-metal-based nanomaterials have shown great potential in cancer therapy due to their intensive near-infrared absorption, excellent photothermal conversion efficiency, strong X-ray attenuation, and magnetic properties. Functional polymers are usually introduced via one-step or multistep methods to further endow these nanomaterials with great biocompatibility and physiological stability. Polymer-decorated transition metal BNMs can be used in multimodal imaging diagnosis and cancer therapy [77]. Magnetic NPs with properly functionalized surfaces can be physically and chemically stable, biocompatible, and environmentally safe. Surface-coating strategies are required to facilitate the application of magnetic NPs in nanomedicine. In the biomedical applications of synthetic nanocomposites, one of the most important properties these materials should possess is high biocompatibility (or low toxicity) when exposed to cells, tissues, or organisms [78]. The authors of [79] considered a new general approach for devising multifunctional magnetic NPs containing different layers, i.e., core-magnetic NPs, with a middle layer containing different therapeutic and imaging agents and an outer layer containing different functional groups (peptides, antibodies, and aptamers) for targeting tumor tissues and for achieving simultaneous sensing, imaging, and therapy. Poon et al. [80] synthesized hybrid metal oxide–peptide amphiphile micelles consisting of an iron oxide or manganese oxide core and a fibrin-binding peptide self-assembling into 20–30 nm spherical NPs. These hybrid NPs were found to be biocompatible with human aortic endothelial cells *in vitro*, and they bound to human clots three to five times more efficiently than their nontargeted counterparts. Lee et al. [81] revealed four main routes of the cytotoxic mechanism of AgNPs (Figure 5).

Next, we dwell in more detail on the consideration of three groups of BNMs that differ in their compositions of functional elements (fillers).

## 3. Magnetic Biomimetic Nanomaterials

The biomimetic synthesis approach has been successfully used in the synthesis of magnetic materials ranging from ferrimagnetic Fe_3_O_4_ (found in magnetotactic bacteria (MTB)) to hard magnetic alloys (such as FePt and CoPt). The use of protein cages for the synthesis of magnetic BNMs provides a number of unique advantages since such structures are of biological origin and, at the same time, are suitable for scalable production and can also be modified using chemical or genetic engineering approaches to give them a specific chemical or structural functionality [82,83]. Ten years ago [64], the overwhelming majority of publications on the synthesis of magnetic NPs were devoted to physical and chemical methods, while only a small part of the works was related to biological methods (using protein constructs, bacteria, or fungi). In recent years, the development of the technology of magnetic BNMs has made it possible to reproduce chains of magnetic NPs similar to those formed in MTBs in a laboratory. Various biomimetic approaches to the synthesis of such chains with optimal properties for biomedical use have been demonstrated [84].

**Figure 6:**
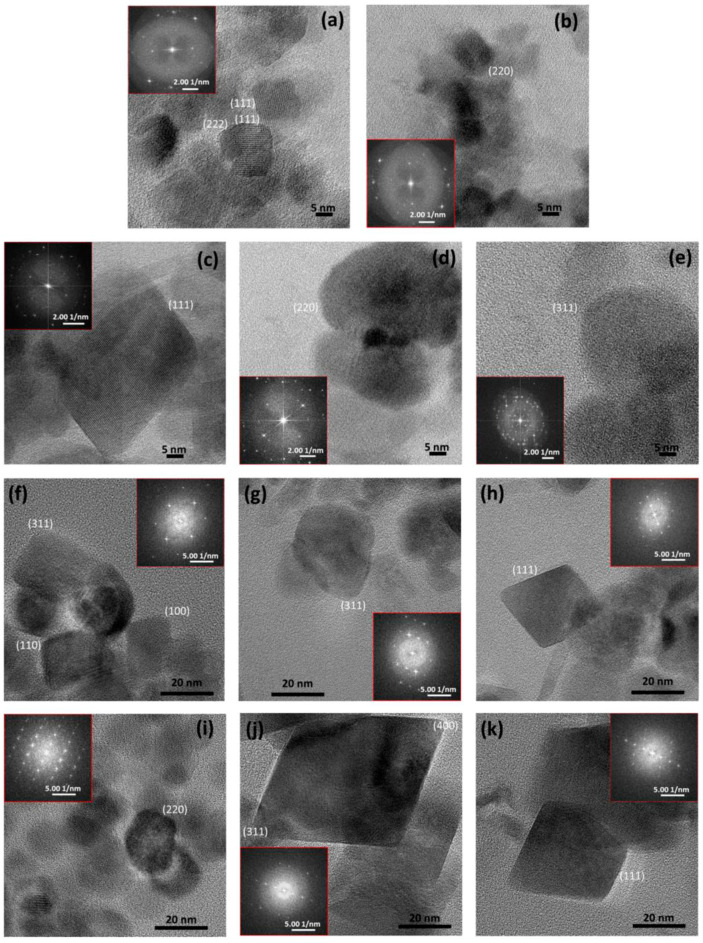
HRTEM images of different NPs: (**a**,**b**) inorganic magnetite NPs, (**c**–**e**) MamC magnetite NPs, (**f**–**h**) Mms6 magnetite NPs, and (**i**–**k**) Mms6-MamC-mediated NPs. Selected areas of electron diffraction are shown for each sample. (Reprinted from [32], license CC BY 4.0.)

One of the studied types of magnetic BNMs is polymer-coated and nonpolymer-coated magnetite NPs obtained using magnetosome-associated MTB proteins [32]. When implementing this method, the biomineralization of Fe_3_O_4_ is carried out in an oxygen-free aqueous solution containing the recombinant magnetosome protein, usually for 30 days. Such magnetic NPs can be used for photothermia or magnetic hyperthermia, chemotherapy, and enzyme immobilization [32,48,85,86,87]. It was experimentally established in [32] that, when two magnetosome proteins (MamC and Mms6) were used for biosynthesis at once, it was possible to obtain magnetic NPs that were close in shape and size to magnetosome crystals [88]. Electron microscopic images of magnetic NPs obtained chemically and using the MamC and Mms6 proteins are shown in Figure 6 [32].

Another option for magnetic BNMs is the use of engineered structures based on bacterial magnetosomes. In this case, magnetosomes are isolated from MTB cells and subjected to purification, followed by biotinylation or encapsulation in an inorganic shell. Such magnetic NPs can be used for contrast enhancement in magnetic resonance imaging, magnetic particle imaging, and magnetic hyperthermia [44,89].

**Figure 7:**
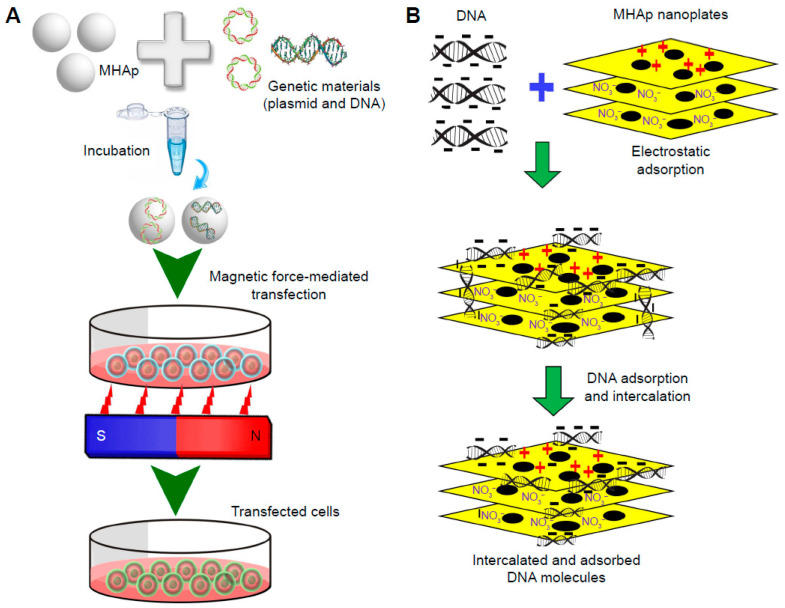
Magnetofection for gene delivery: (**A**) schematic representation of the process and (**B**) schematic illustration of DNA loading into lamellar magnetic hydroxyapatite (MHAp) nanoparticles for nucleic acid delivery. (Reprinted from [35], license CC BY 3.0.)

The construction of magnetic BNMs based on magnetite NPs coated with hydroxyapatite (HAP) is described. The preparation of such constructs is usually carried out by introducing a liquid HAP precursor into a solution containing preliminarily formed magnetite cores. The biomedical applications of such magnetic NPs can be magnetic hyperthermia, the creation of magnetic scaffolds for bone tissue restoration, and the delivery of genetic material [35,51], as shown in Figure 7.

Integration in a single biomimetic matrix (for example, SiO_2_) of magnetic NPs and the active substance is possible. Such BNMs can be obtained by the hydrolysis of tetramethyl orthosilicate in the presence of preliminarily obtained magnetite NPs and an enzyme solution. A possible area of application is the development of biocatalysts for targeted enzyme prodrug therapy [37].

A summary of the literature data on the structure, synthesis, and applications of magnetic BNMs is given in Table 1.

An analysis of the methods for the synthesis of magnetic BNMs allowed us to draw the following conclusions. All the methods considered in Table 1 could be conditionally divided into two groups: methods based on the use of previously obtained or commercial magnetic NPs and methods that included the process of obtaining magnetic NPs. The first group of methods included the introduction of precursors of the HAP biomimetic component into a colloidal solution of magnetic NPs [35,51], acrylamide and ethylene glycol dimethacrylate copolymerization in the presence of S-naproxen on silica-coated Fe_3_O_4_ NPs [90], preliminarily prepared Fe_3_O_4_ NP incubation in an alkaline dopamine solution [93], and the self-assembly of Fe_3_O_4_ NPs coated with a hydrophilic polymer into 1D nanochains in water [95].

Methods of the second group, from our point of view, were more promising due to the ability to create a material structure similar to natural analogues at the level of individual atoms. They included the biosynthesis of magnetic NPs from oxygen-free solutions containing recombinant MamC in anaerobic conditions [32,48,85,86,87], magnetite biomineralization using PEGylated human ferritin NPs [52], and the silica encapsulation or biotinylation of isolated bacterial magnetosomes [44,89]. Thus, the methods of the second group made it possible to achieve a combination of the advantages of biogenic magnetic NPs (magnetosomes) and their synthetic counterparts, minimizing the disadvantages of both.

Despite the long history of the development of magnetic BNM technology [68], this class of materials is not among the most widespread group of biomimetics.

## 4. Metal and Metal Oxide Biomimetic Nanomaterials

The physical and chemical properties of metal and metal oxide NPs can be controlled by choosing their micro- or nanoenvironments during synthesis. Some of the best-known platforms currently in use, in particular for the biomimetic synthesis of metal oxides, include ferritin, viral capsids, or bacterial cells. These biotemplates provide strictly defined conditions for the formation of NPs and, thus, their narrow distribution in shape and size [96]. In addition, such synthetic approaches are of interest due to the development of environmentally friendly NP technology. One of the most discussed approaches is the synthesis of metal NPs using organisms, of which plants are considered the best candidates and are suitable for the scalable production. The growing advantages of using plants and herbs for the biomimetic synthesis of metal NPs has prompted scientists to search for the mechanisms of metal ion biological reduction and to study in more depth the mechanisms of metal NP synthesis in plants [97]. Obtaining Ag NPs for the purpose of therapy for multiresistant pathogenic microorganisms can be carried out using plant components. Thus, the preparation of such NPs by the reduction of silver nitrate in an aqueous solution with the addition of extract of *Musa balbisiana* or *Phlomis bracteosa* seedlings, as well as *Saraca indica* leaves, as a reducing agent, has been described [55,98,99].

**Figure 8:**
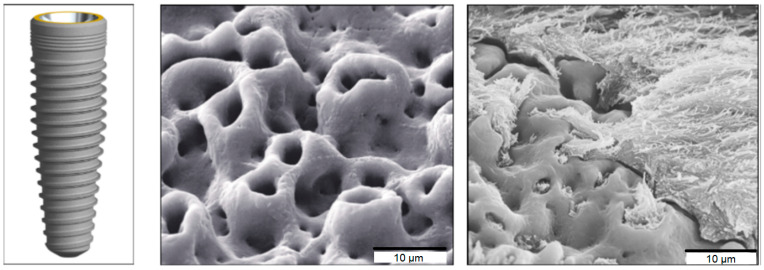
Surface features and scanning electron micrographs of a TiUnite dental implant surface. (Reprinted from [38], license CC BY 4.0.)

The development of biomimetic methods for the synthesis of metallic nanomaterials plays an important role in dentistry. In recent years, various approaches have been used to modify the topographical or chemical properties of traditional implant surfaces in order to improve the adhesion of the implant material to bone cells. Changing the surface of a titanium implant makes it possible to stimulate the bone tissue, minimizing the period of osseointegration and ensuring the good transfer of occlusal mechanical loads from the implant to the bone [38] (Figure 8).

A titanium base with a nanostructured calcium phosphate coating obtained by introducing HAP ceramic particles into a plasma jet directed at the treated Ti surface can be used in dentures [100].

One of the areas of application of metallic BNMs is the detection and elimination of natural and synthetic pollutants. Gold, silver, and bimetallic Ag-Au NPs for the purpose of creating sensors for biological substances and research in the field of nanotoxicology were obtained using a biomimetic method in an aqueous solution of gelatin with the successive addition of silver nitrate or hydrogen tetrachloroaurate [39].

**Figure 9:**
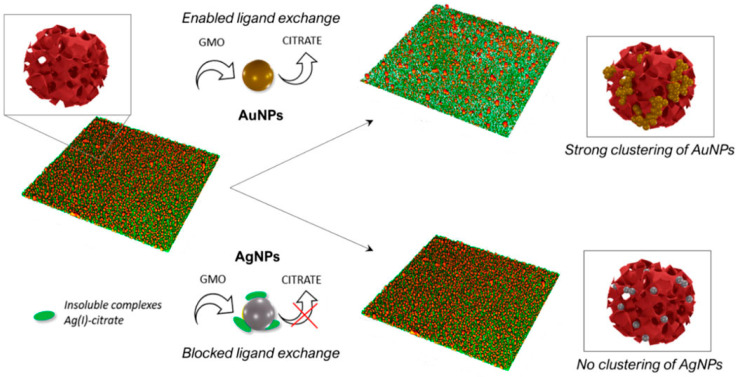
Schematic representation of the mechanism and final outcomes of the interaction of Au NPs and Ag NPs with a water dispersion of cubosomes and solid-supported films of cubosomes. (Reprinted from [34], license CC BY 4.0.)

The new, proposed biomimetic methods for obtaining Au NPs and Ag NPs include the use of “cubosomes”, model cubic nanostructures based on lipid membranes. Such BNMs are obtained through incubating preformed Au or Ag NPs with cubosomes in an aqueous solution [34] (Figure 9). The purpose of BNMs based on cubosomes is the targeted delivery of drugs.

Another important direction in the development of BNM technology for use in sensors of biological substances and oncology is the production of metal–organic frameworks (MOFs) [58,101]. Pt NPs could be synthesized using MOFs prepared using Fe(III)tetra(4-carboxyphenyl)porphine chloride as a template [101]. More complex structures, porphyrin Zr-MOFs coated with a cell membrane for the purposes of antiangiogenesis and photodynamic therapy in oncology, were obtained using solution chemistry followed by the deposition of manganese oxide from KMnO_4_ and camouflaging with a cell membrane [58]. Composite sponges of stearic acid do not exhibit good adsorption capacity as compared to MOFs and carbonaceous-material-based sponges. Moreover, they have complex synthesis procedures and difficult oil recovery methods. Azam et al. [102] fabricated a composite sponge with hydrophobicity as high as that of stearic acid sponges, as high an oil absorption capacity as that of MOF-based sponges, and excellent reusability of up to 10 cycles utilizing the inherited properties of MOFs (i.e., high surface area, controllable pore size, and chemical functionality at the molecular level), the nonwetting and superhydrophobic property of stearic acid, and the regular 3D skeleton of a highly porous polyurethane sponge.

The authors of [103] explored the use of MOFs formed using the self-assembly of metal ions and organic building blocks to safeguard collagen integrity in the functional dentin matrix. They demonstrated that collagen fibrils (from demineralized human dentin) could induce the biomimetic growth of MOF crystals as protective coatings to strengthen and stabilize the fibrils. Chen et al. [104] focused on the recent progress in biomimetic MOF catalysts. The authors summarized principles and strategies for the design and synthesis of biomimetic MOF catalysts, discussed structure-related catalytic properties, and particularly addressed important factors, including (1) active sites, (2) microenvironment, (3) transmission channels, and (4) co-catalytic sites, on the distinct catalytic properties of biomimetic MOFs. In addition, some examples have been given to illustrate the synergistic catalysis between multiple factors that are closely related to enzymatic catalysis. It is highly desirable, yet remains challenging, to achieve the synergy of specific functions between MOF host and guest species. Cheng et al. [105] obtained a novel MOF composite biomimetic structure inspired by a natural multienzyme system and based on the co-encapsulation of glucose oxidase and L-arginine into Cu-MOFs with Fenton-like catalytic activity to achieve a synergistic antibacterial effect. A facile strategy to prepare a biomimetic cascade reaction system by combining the advantages of enzyme immobilization and biomimetic catalysis in a one-pot reaction system based on a hierarchically porous metal–organic framework was reported [106].

**Figure 10:**
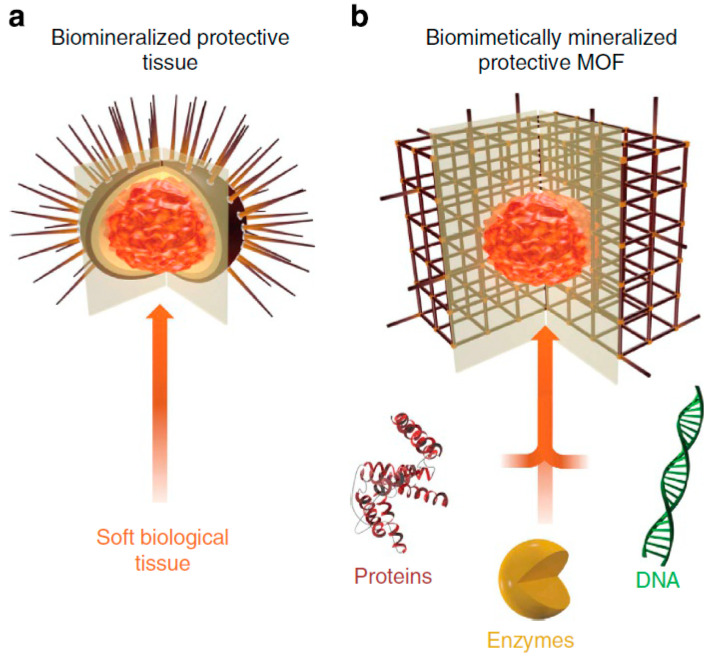
Schematic illustration of biomimetically mineralized metal–organic framework (MOF). (**a**) Schematic of a sea urchin, a hard, porous, protective shell that is biomineralized by soft biological tissue. (**b**) Schematic of an MOF biocomposite showing a biomacromolecule (for example, protein, enzyme, or DNA) encapsulated within a porous, crystalline shell. (Reprinted from [108], license CC BY 4.0.)

MOFs are ideal candidates for building biomimetic systems because their uniform cavities can generate a high density of biomimetic active centers. In addition, thermally and chemically stable MOFs can be built with a variety of metal clusters. Furthermore, the channels of MOFs provide confined pockets, which protect the catalytic centers and enhance substrate specificity [107]. Liang et al. [108] reported that a wide range of biomacromolecules, including proteins, DNA and enzymes, could efficiently induce MOF formation and control the morphology of the resultant porous crystal via a biomimetic mineralization process under physiological conditions. The authors demonstrated that the biomimetic mineralization of MOFs formed a nanoporous shell, which encapsulated the biomacromolecules and afforded unprecedented protection from biological, thermal, and chemical degradation with the maintenance of bioactivity (Figure 10).

Singh et al. [109] proposed a protective framework offering thermal stability especially for vaccines wherein additives and stabilizers alone may not be sufficient. The authors applied an MOF biomimetic mineralization technique for live viral vaccine encapsulation. This is a ubiquitous stabilization technology that is a simple, rapid, and one-step scalable approach using cost-effective ingredients and ambient aqueous conditions. Water-stable Fe(III)-based MOFs, which exhibit intrinsic peroxidase-like activities, catalyzing the oxidation of 3,3′,5,5′-tetramethylbenzidine and o-phenylenediamin when H_2_O_2_ served as the oxidant, were constructed and characterized [110]. Their catalytic performances strongly relied on pH value, temperature, catalyst dosage, and H_2_O_2_ concentration. Compared to natural enzymes, peroxidase mimetics had the advantages of low cost, ease of preparation and storage, greater resistance to biodegradation, and less vulnerability to denaturation. The presented results could provide a possibility of building MOF-based platforms as enzymatic mimic catalysts and could facilitate their utilization in immunoassays and biotechnology. With a reactivity that mimicked the activity of an enzyme, the MOF-based material also catalyzed the isotopic exchange of oxygen between water and carbon dioxide. Wright et al. [111] provided convincing evidence that the metal nodes in MOFs had high structural fidelity with respect to the active sites of enzymes.

**Figure 11:**
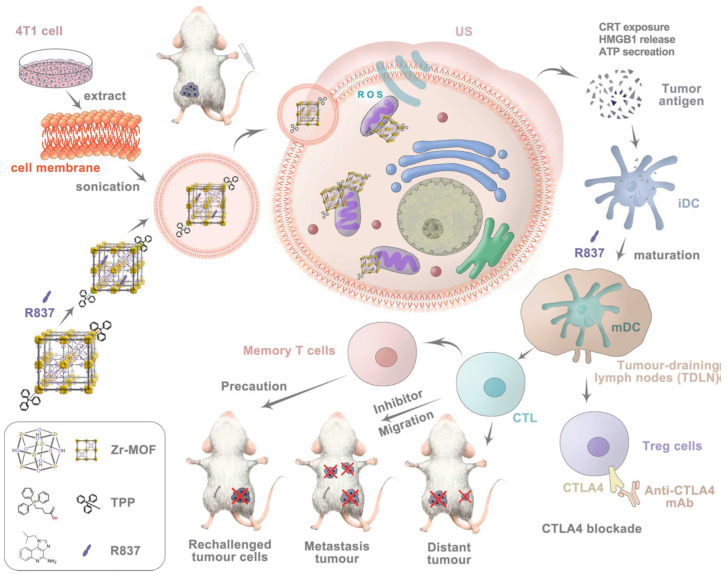
Schematic illustration of mechanism of mitochondria-targeted cancer cell membrane biomimetic metal–organic framework mediated sonodynamic therapy and immune checkpoint blockade immunotherapy. (Reprinted from [115], license CC BY 4.0.)

Ma et al. [112] designed a renewable, inexpensive, and easy-to-assemble filter prepared by growing an MOF on wood, followed by high-temperature carbonization. The proposed filter demonstrated a superior dye removal performance and was expected to replace commercial activated carbon and to be successfully applied in practical wastewater treatment fields. The effect of a peptide or protein sequence on the biomimetic mineralization of zeolite imidazolate frameworks (ZIFs), as well as a general approach to make peptide@ZIFs via a biomimetic mineralization process, was investigated [113]. The reaction rate of peptide@ZIF formation was compared for different types of peptides with different isoelectric points. Wang et al. [114] developed a new MOF–poly(amidoxime) composite membrane with a permeable macroporous structure, good mechanical properties, and high efficiency for uranium extraction from seawater. Cancer-cell-membrane-coated, triphenylphosphonium-decorated nano-MOF constructs were synthesized to achieve homologous mitochondria-targeted sonodynamic therapy efficacy in combination with simultaneous delivery of the Toll-like receptor agonist R837 as an immune adjuvant [115] (see Figure 11).

The above, as well as other examples of BNMs based on metal particles and metal oxides, are summarized in Table 2.

Comparing the summary data in Table 2 with those presented earlier in Table 1, one can draw attention to the predominantly different synthesis techniques for obtaining BNMs of the two considered groups, despite the wide variety in the approaches to synthesis proposed to date. In the case of BNMs based on magnetic particles, the biomineralization of magnetite and the use of preformed magnetic NPs are most commonly used.

For classical, well-developed BNMs based on metal particles and metal oxides, reduction from precursor solutions is most often used as a method. Nevertheless, in recent years, a newer class of BNMs including metal components has been developed rapidly in the form of MOFs. The wide perspectives of this class of materials are mainly due to its multifunctionality, including antiangiogenesis and photodynamic therapy [58]; biosensing [101]; sonodynamic therapy and immune checkpoint blockade immunotherapy [115]; protection from biological, thermal, and chemical degradation with the maintenance of bioactivity [108]; antibacterial therapy; and biomimetic catalysis [106]. Nevertheless, the technology of MOFs and, especially, composite BNMs containing MOFs is still challenging [104].

This may be due, from our point of view, not only to the chemical natures of the oxide and metal components of BNMs, but to the differences in the main claimed applications of these materials, as shown later in Section 5.

## 5. Organic, Ceramic, and Hybrid Biomimetic Nanomaterials

BNMs based on organic and ceramic components are by far the most widely represented compared to the two groups of BNMs described above. When obtaining BNMs based on organic components, molecular mechanisms for constructing proteins and peptides can be used, which control the synthesis of nanosized objects and the self-assembly of systems of multifunctional materials of a higher order. For this, solid-binding peptides are used, and the formation, assembly, and organization of functional nanoobjects are controlled [119]. Protein- and peptide-based biomimetic mineralization has been shown to be an efficient and promising strategy for the synthesis of magnetic and ceramic NPs. Proteins or peptides can bind metal ions through various metal-binding sites, such as N-terminal amines or multi-metal binding sites, and thus, their unique structures allow them to be used as NP growth templates [120]. A new class of biomimetic polymers is peptoids (or poly-N-substituted glycines), which are perceived due to highly efficient synthesis, high chemical stability, resistance to enzymatic hydrolysis, and biocompatibility. Due to their properties, amphiphilic peptoids are considered as customizable building blocks for obtaining BNMs with hierarchical structures and specified functionalities [121].

The development of ceramic BNM technology is largely aimed at creating scaffolds that mimic natural tissue and reproduce its properties, such as composition and microstructure. In particular, for bone tissue engineering, this is most often achieved by fabricating highly porous ceramic structures that resemble cancellous bone. These scaffolds possess many important properties, including biological activity, space for cells, and ingrowth of new tissues, as well as acceptable mechanical strength. However, when using such scaffolds, the new tissue is often disorganized and has poor mechanical properties [122]. The formation of three-dimensional tissue in the laboratory is highly dependent on the biomimetic environment, engineered extracellular matrix, and cell type, as well as biologically active components. Scaffolds should resemble the structural and biochemical features of the natural extracellular environment in order to support and control cell migration and growth [123]. In addition to being used in solving tissue-engineering problems, biomimetic ceramic scaffolds are often loaded with various therapeutic molecules to increase their biological effectiveness [124]. The peptide-mediated remineralization of artificially induced tooth lesions and the characteristics of orientation, formation, and composition of a newly formed enamel-like apatite layer were experimentally evaluated. Amelogenin was used as a model peptide [41,125].

A biomimetic design of light, high-temperature ceramics based on functionally differentiated structures was proposed. Such structures can be found in nature, in particular, on cross-sections of the bones, teeth, and stems of many plants, such as bamboo. Originally conceived for spacecraft heat shields, functionally differentiated structures are finding more and more applications in other areas, including biomedicine [126]. Researchers are attracted by the hierarchical structure of nacre, which contains at least six levels of hierarchy in the size range from a few millimeters to ~100 nm. In addition, the biomineralization of nacre is carried out by the self-assembly of elementary units under mild conditions at ambient temperature and either neutral or physiological pH, always in an aquatic environment [127]. For the production of bioactive orthopedic biomaterials using biomimetic nanocrystalline apatites, several approaches have been used, among which are *in situ* formation, which allows obtaining a highly reactive structure, and low-temperature molding [128]. Biomimetic ceramics of complex shapes can be obtained on the basis of self-forming, two-layer microstructures based on aluminum oxide. Such a material is produced by the repeated coagulation of a suspension of Al_2_O_3_ NPs with a ferrofluid under the action of a magnetic field, followed by high-temperature annealing [36].

A promising object of research is biomimetic catalysts based on chiral nanomaterials, which can cause the same biological effects as natural catalysts and has demonstrated high efficiency in biological applications [129]. The biomimetic synthesis of materials and the template growth of inorganic or hybrid networks using self-assembling, hybrid, organic–inorganic interfaces are also of considerable interest to researchers since artificially obtained hybrid materials lie at the junction of the organic, inorganic, and biological worlds [130].

Fatima et al. [9] obtained a composite based on bacterial cellulose waste modified with bioactive plant extract. The material showed high bactericidal activity against *S. aureus* and produced a clear inhibition zone, whereas negligible activity was observed against *E. coli*, indicating its bactericidal activity mainly against Gram-positive bacteria. Overall, their study illustrated that there is a huge potential for developing valuable biomaterials from food wastes and utilizing their liquid-holding capabilities for value-added applications in the medical and pharmaceutical fields. Chen et al. [131] reported a bamboo–nigrosine–poly(N-isopropylacrylamide) composite hydrogel actuator capable of complex deformations and near-infrared light response based on bamboo sheets. The simple and rapid ultraviolet *in situ* polymerization method closely connected bamboo and hydrogel so that the composite hydrogel actuator was difficult to peel off during the actuation process. In addition, the composite hydrogel actuator exhibited diverse shape deformations because of the natural anisotropy of the bamboo sheets. A composite thermoresponsive actuator with multifunctional actuation upon an alternative magnetic field was fabricated [132]. Fe_3_O_4_-NP-embedded thermoplastic polyurethane film and a photo-crosslinkable electrospun fibrous poly(N-isopropylacrylamide) mat formed the bilayer actuator.

**Figure 12:**
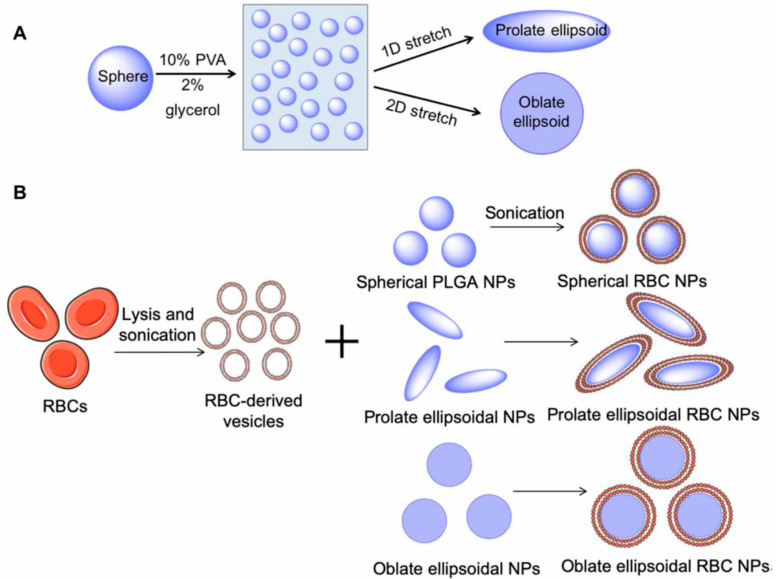
Schematic of anisotropic nanoparticle fabrication and RBC membrane coating. (**A**) Spherical PLGA nanoparticles (NPs) were synthesized and cast onto a thin plastic film of 10% polyvinyl alcohol (PVA) and 2% glycerol. Particles were then stretched under heat in one and two dimensions (2D) to generate prolate and oblate ellipsoidal particles, respectively. (**B**) RBCs underwent hypotonic lysis and were then sonicated to generate sub—200 nm vesicles. RBC-derived vesicles were then coated on PLGA nanoparticles of all shapes under sonication. (Reprinted from [33], license CC BY 4.0.)

The removal of systemically administered bacterial toxins could be accomplished using anisotropic polymeric NPs camouflaged with an erythrocyte membrane. Spherical particles of poly(lactic acid-glycolic acid) (PLGA) were immobilized on a film of polyvinyl alcohol and glycerol mechanically stretched to change the shape of the NPs from isotropic to anisotropic, and then erythrocyte membranes were deposited with an ultrasonic treatment [33] (Figure 12).

Important areas of application for polymeric NPs camouflaged by cell membranes are bioimaging, phototheranostics, and the development of nanovaccines. NPs of PLGA with tumor cell membrane envelopes could be obtained by depositing the drug in PLGA using precipitation from a solution, followed by coating with a cell membrane [45].

**Figure 13:**
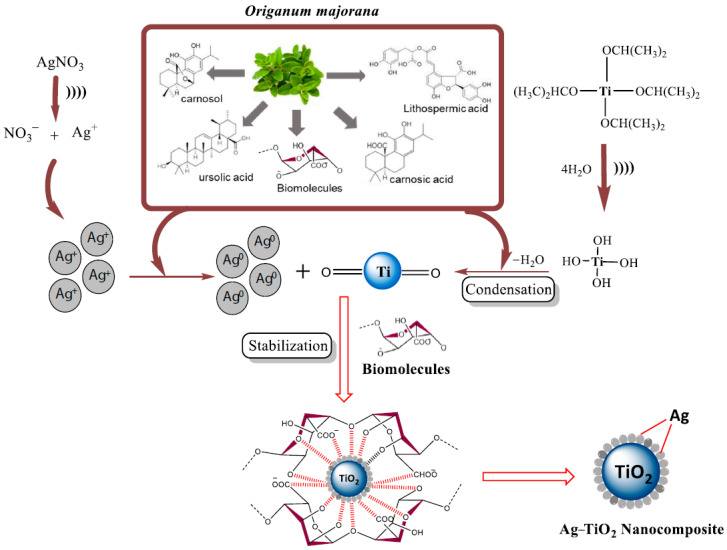
Plausible mechanism for the formation of Ag–TiO_2_ NCs using *Origanum majorana* leaf extract. (Reprinted from [40], license CC BY 4.0).

As well as metal NPs (see Section 4), more complex NPs can be obtained biomimically using plant extracts as sources of natural reducing agents and stabilizers. Thus, Ag–TiO_2_ NPs were obtained through sonochemical synthesis using *Origanum majorana* leaf extract as a restoring and stabilizing agent, which could be used for antibacterial and antioxidant therapy [40] (Figure 13).

Much attention from BNM researchers has been paid to works in the field of dentistry, primarily to the restoration of demineralized tooth enamel. One of proposed approaches is amelogenin-containing chitosan hydrogel [133,134]. The preparation of this material is carried out by the mixing of a solution of chitosan, calcium chloride, and recombinant porcine amelogenin, followed by mechanical agitation. Li et al. used HAP crystallized with a polyamidoamine (PAMAM) dendrimer template [135,136,137]. The treated tooth enamel was placed in a solution containing CaCl_2_, KH_2_PO_4_, and PAMAM dendrimers modified with carboxyl groups.

An important task in the preparation of BNMs based on NPs of different natures (oxide, metal, polymer, and semiconductor) is the possibility of experimental evaluation of the efficiency of coating particles with a cell membrane obtained from tumor cells or normal human cells (macrophages, platelets, or erythrocytes).

The use of a fluorescence-quenching assay to assess the integrity of cell membranes covering BNMs has been proposed. Using this approach, the authors of [138] measured and compared the degree of membrane coverage of BNMs and found that the approach used today did not provide adequate integrity of the cell membrane. It was demonstrated that such partially coated NPs were able to enter tumor cells through a cooperative mechanism based on NP aggregation.

Another large group of problems solved through the use of BMNs and, in particular, materials based on a natural or artificial phospholipid membrane encapsulating a drug agent is the therapy of inflammatory diseases, including atherosclerosis. The potential use for anti-inflammatory therapy of leukocyte-based biomimetic NPs obtained by combining phospholipids and leukocyte membrane proteins followed by incubation with binding antibodies has been described. Moreover, the process can be carried out both in a batch [109] and in a continuous flow (microfluidic) [67] reactor. Such biomimetic nanovesicles are called leukosomes [67,139].

In addition to leukocytes, macrophages can serve as a basis for constructing BNMs intended for the treatment of atherosclerosis. The authors of [46] proposed a construct based on a macrophage membrane in which polymeric NPs capable of responding to reactive oxygen species (ROS) were camouflaged. The synthesis was carried out by applying a shell of cell membranes to preliminarily obtained polymeric NPs by extrusion through a polycarbonate membrane with a pore size of 400 nm. The resulting ROS-responsive biomimetic NPs had a small hydrodynamic size with a negative surface charge, retained functional proteins from macrophage membranes, and demonstrated ROS-responsive drug release. Due to cell membrane camouflage, the NPs were able to effectively avoid capture by macrophages and target inflammatory endothelial cells. In addition, such BNMs have demonstrated the ability to inhibit the proliferation of macrophages and smooth muscle cells *in vitro* without significant cytotoxicity.

A summary of the above and additional examples of BNMs based on polymer, ceramic, and mixed components is shown in Table 3.

As one of the most intensively developing classes of nanomaterials, hybrid BNMs have attracted considerable attention, especially toward the development of technologies for their formation. By analyzing the data presented in Table 3, it was concluded that there were two main groups of hybrid BNMs: the first one included BNMs based on biomimetic nanoceramics, and the second one was based on the use of biogenic or artificial cell membranes with organic (polymeric) or inorganic functional cores. We believe that both groups of BNMs can be considered as equally prospective for biomedical use.

Biomimetic nanoceramics have been obtained using various methods, including Al_2_O_3_ NP repeated coagulation with ferrofluid under a magnetic field following sintering [36], nonstoichiometric silicon nitride (Si_x_N_y_) deposition on both sides of a silicon wafer using low-pressure chemical vapor deposition [142], self-assembly of a layered chitosan–maleic acid matrix followed by monetite mineralization and transformation to HAP [147], and alumina powder addition to a chitosan solution followed by alginate dissolution and genipin (cross-linking agent) addition. The main proposed application of these biomimetic nanoceramics has been tissue engineering for orthopedic and dentistry purposes.

Membrane-camouflaged NPs have usually been synthesized, for example, via drug-encapsulating PLGA prepared via nanoprecipitation with a consequent coating with cancer cell membranes [45], RBC hypotonic treatment and extrusion followed by mixing with PLGA NPs via extrusion through a porous membrane [66], stretching of spherical PLGA NPs immobilized on a PVA-glycerol film followed by sonication-assisted coating with ultrasound-derived RBC membranes [33], and biodegradable NPs conjugated with (3-aminopropyl)triethoxysilane followed by incubation with proteolipid solution [144]. Such BNMs have been mainly proposed for the development of drug delivery carriers.

## 6. Summary

**Figure 14:**
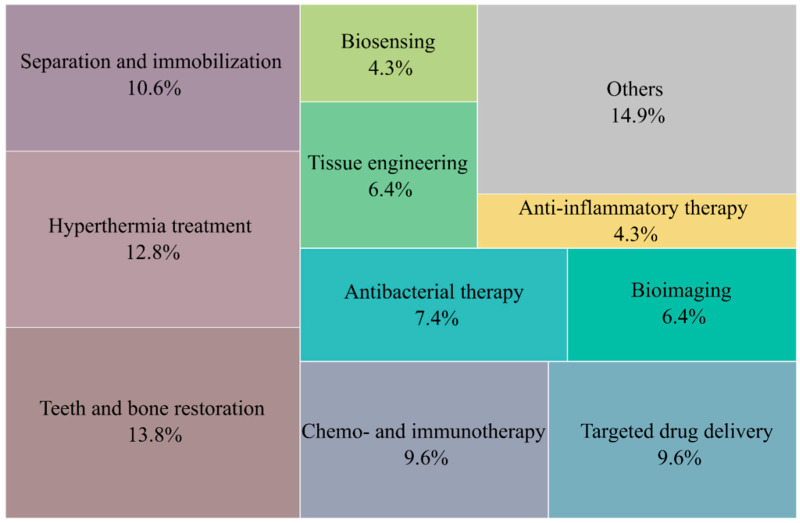
Main applications of biomimetic nanomaterials.

The scientific literature reviewed was summarized based on the application areas of BNMs of the three considered main groups. The distribution of the applications of BNMs indicated in Table 1, Table 2 and Table 3 is shown in Figure 14. The diagram shows that biomedical applications of such materials are currently predominant.

The main applications included tasks related to the use of BNMs inside the human body: the restoration of damaged tooth enamel and the restoration of bone tissue; magnetic and infrared hyperthermia; chemotherapy and immunotherapy for oncological diseases; the development of new drugs for targeted therapy; and antibacterial and anti-inflammatory therapy and bioimaging. It can be assumed that, due to the high technological complexity and production costs of BNMs, their practical uses are justified, first of all, to treat socially significant infectious and noninfectious diseases.

From our point of view, BNM technologies based on the uses of natural (native), genetically modified, or completely artificial phospholipid membranes are currently of the highest demand in biomedical applications. Such constructs are able to combine the physicochemical and biological properties of biogenic prototypes (for example, bacterial magnetosomes) with high biocompatibility (due to the absence of microbial toxins), economic availability (expensive biotechnological synthesis is not required), and scalability for the production of synthetic nanomaterials. The most frequently used inorganic bases (cores) for obtaining such biomimetic nanostructures include (i) magnetite, (ii) hydroxyapatite, (iii) Au and Ag, and their combinations due to in-demand applications in the fields of drug delivery, tissue engineering, and antibacterial therapy. The most frequently obtained organic and hybrid cores of BNMs include (i) metal–organic frameworks, (ii) poly(lactic acid-glycolic acid), and (iii) polyamidoamine dendrimers due to applications in nanovaccines, anti-inflammatory therapy, and biocatalysis.

Finally, by analyzing the most recent literary sources, it was possible to list the main challenges that stand in the way of the development of BNM technology: (i) there remains much room for the improvement of BNMs in terms of their stability and biocompatibility since BNMs have been used only in animal studies, but they have not been widely used in clinical practice [22]; (ii) understanding the energetic contributions that rule interactions at the nano–bio interface (i.e., where NPs meet biological barriers, specifically cell membranes) is very complex due to the high compositional heterogeneity of biomembranes and the intrinsic variability of the biological environment [34]; (iii) the clinical application of BNMs encounters complex fabrication processes, unsuitable large-scale production, low yields, and difficult preservation [24]; (iv) the utilization of biosynthesis (e.g., engineered bacteria) requires scaled-up manufacturing, dose determination, and potential biosafety studies [23]; (v) the processing of biomaterials (e.g., bioprinting) that incorporate living cells is still very challenging, especially considering that the production of mechanically rigid and insoluble substrates usually requires nonbiocompatible processes, such as chemical cross-linking or sintering [65].

## Figures and Tables

**Table 1:** Magnetic biomimetic nanomaterials: structure, synthesis, and applications.

Composition	Synthesis Technique	Declared Applications	Refs.
HAP ^1^-coated magnetite NPs	HAP precursors added into a solution containing iron oxide NPs	Magnetic hyperthermia, magnetic scaffold for bone tissue regeneration	[35,51]
Dextran-coated magnetite NPs modified with a protein and a mimetic ligand	Ligand directly synthesized on dextran-coated particles	Magnetic separation of biomolecules	[50]
MamC-mediated biomimetic Fe_3_O_4_ NPs with or without polymer coating	Biosynthesis from oxygen-free solutions containing recombinant MamC in anaerobic conditions for 30 days	Photothermia, chemotherapy, magnetic hyperthermia, immobilization of enzymes	[32,48,85,86,87]
Fe and Fe_3_O_4_ NPs	Microbial preparation of FeS and Fe_2_O_3_ using *Aspergillus niger* YESM 1, followed by physical process at supercritical conditions	Magnetic resonance imaging, magnetic hyperthermia	[49]
PEGylated magnetoferritin NPs with magnetite core	Magnetite biomineralization using PEGylated human ferritin NPs	Magnetic resonance imaging	[52]
Acrylamide-based biomimetic magnetic NPs	Acrylamide and ethylene glycol dimethacrylate copolymerized in the presence of S-naproxen on silica-coated Fe_3_O_4_ NPs	Separation of chiral drugs	[90]
Biomimetic silica entrapping Fe_3_O_4_ NPs and horseradish peroxidase	Tetramethyl orthosilicate hydrolysis in the presence of Fe_3_O_4_ NP suspension and horseradish peroxidase solution	Biocatalyst for direct enzyme prodrug therapy	[37]
Fe_3_O_4_ NPs	Copolypeptide-promoted Fe_3_O_4_ NP biomimetic mineralization	Separation technology, magnetic resonance imaging	[91]
Cell-membrane-camouflaged Fe_3_O_4_ NPs	Cell membrane adsorption onto silica-coated Fe_3_O_4_ or onto drug-loaded mesoporous Fe_3_O_4_ NPs	Drug targeting, cancer immunotherapy	[54,92]
Biomimetic magnetic silk scaffolds	Magnetic NP diffusion into silk fibroin protein via dip-coating	Tissue engineering, magnetic hyperthermia	[53]
Engineered bacterial magnetosomes	Silica encapsulation or biotinylation of isolated bacterial magnetosomes	Magnetic particle imaging, magnetic resonance imaging, magnetic hyperthermia	[44,89]
Polydopamine-coated Fe_3_O_4_ NPs	Preliminarily prepared Fe_3_O_4_ NP incubation in an alkaline dopamine solution	Immobilization of enzymes	[93]
Biodegradable polylactide-based Fe_3_O_4_ NPs	Modified emulsification–solvent evaporation method	Degradation pattern study of NP formulations	[94]
Magnetosome-like ferrimagnetic iron oxide nanochains	Self-assembly of Fe_3_O_4_ NPs coated with hydrophilic polymer into 1D nanochains in water	Post-stroke recovery	[95]

^1^ Hydroxyapatite.

**Table 2:** Biomimetic nanomaterials based on particles of metals and metal oxides and areas of their application.

Composition	Synthesis Technique	Declared Applications	Refs.
Au, Ag, and Ag–Au bimetallic NPs	Biomimetic synthesis in aqueous gelatin solution with consequent addition of AgNO_3_ and HAuCl_4_	Biosensing, nanotoxicology	[39]
Au and Ag NPs attached to model lipid cubic phase membranes	Incubation of preliminarily obtained Au or Ag NPs and cubosomes in aqueous dispersions	Cubosome-based targeted drug delivery	[34]
Electrochemical sensor based on Au-NP-imprinted polymer	Surface modification of metal electrode with 2-aminothiophenol and preliminarily obtained Au NPs, followed by electropolymerization	Organic pollutant detection	[116]
Ag NPs	Biological reduction of AgNO_3_ in aqueous solution with Musa balbisiana or Phlomis bracteosa plantlets or Saraca indica leaf extracts as reducing agents	Multidrug-resistant bacteria treatment	[55,98,99]
Au–Ag NPs attached on silica nanowire support	Silica nanowire formation using cellulose nanocrystals as biotemplates, followed by Au–Ag NP attachment via wet chemical process	Network substrate in surface-enhanced Raman scattering	[61]
Au, Pd, and Pt NPs on biomimetic MXene paper	Spontaneous growth of metal NPs from aqueous precursor solution on Ti_3_C_2_T_x_ paper obtained using vacuum filtration	Flexible bioelectronics	[117]
Au–Pd NPs in amide conjugate structure	Formation of Au–Pd NPs from HAuCl_4_ to PdCl_2_ self-assembled gallic acid amid conjugates	Catalytic degradation of organic pollutants	[56]
Porphyrinic Zr–MOF ^1^ NPs cloaked with cell membrane	Wet chemistry synthesis of Zr–MOFs, followed by MnO_2_-coating in KMnO_4_ solution and cell-membrane-cloaking	Antiangiogenesis and photodynamic therapy	[58]
Peptide-coated Au NPs	Reduction of HAuCl_4_ in aqueous solution of multifunctional peptides	Biosensing, targeting NPs into cells and organelles	[57]
Au nanoplates	Reduction of HAuCl_4_ in aqueous solution using Chlorella vulgaris extract	Near-infrared range hyperthermia	[59]
ZnO, NiO, CuO, Co_3_O_4_, and CeO_2_	Eggshell membrane immersion in metal salt solutions, followed by drying at room temperature and calcination at 750 °C	Removal of NPs from an aqueous environment	[60]
Polydopamine-Ag NP membrane	Treatment of catheter surface with a dopamine solution, followed by AgNO_3_ solution immersion and vacuum-drying	Central venous catheter coating	[118]
Pt-NP-decorated metal–organic framework	Synthesis of Pt NPs templated with MOFs obtained using Fe(III) tetra(4-carboxyphenyl)porphine chloride	Biosensing	[101]
Nanostructured calcium-phosphate-coated Ti	HAP ^2^ ceramic particle injection into a plasma torch and projection on the surface of titanium	Dental implants	[38,100]

^1^ Metal–organic framework; ^2^ Hydroxyapatite.

**Table 3:** Polymeric, ceramic, and hybrid biomimetic nanomaterials and their applications.

Composition	Synthesis Technique	Declared Applications	Refs.
Microstructured Al_2_O_3_ self-shaped bilayers	Al_2_O_3_ NP repeated coagulation with ferrofluid under magnetic field following sintering	Biomimetic complex-shaped ceramics	[36]
Cancer-cell-membrane-coated polymeric NPs	Drug-encapsulating PLGA prepared via nanoprecipitation consequently coated with cancer cell membrane	Bioimaging, phototheranostics, nanovaccines	[45]
ZrO_2_ coated with HAP ^1^–bovine serum albumin composite	ZrO_2_ substrate soaked in albumin and simulated body fluid solution, followed by calcium phosphate nanocrystal precipitation	Orthopedic and dentistry	[140]
Phage–platelet hybrid NPs	Binding of a blood-circulation-prolonging, peptide-modified bacteriophage to platelet membrane NPs derived via a repeated freeze–thaw procedure	Blood-retention-time-prolonging, antibacterial phage therapy	[141]
Ultrathin silicon nitride microporous membranes	Nonstoichiometric silicon nitride (Si_x_N_y_) deposition on both sides of a silicon wafer by low-pressure chemical vapor deposition	Scaffolds for epithelial tissue cell models	[142]
PLGA ^2^ NPs wrapped with MMs ^3^	Mixing of drug-containing PLGA NPs with purified macrophage membranes and following extrusion using a 200 nm polycarbonate membrane	Ulcerative colitis treatment	[143]
Fe_3_O_4_, ZIF ^4^, Au, PLGA, and porous Si coated with cell membrane	Sonication or extrusion coating of various NPs with HeLa, macrophages, platelets, and RBC ^5^ cell membranes	Cancer nanomedicine	[138]
Leukocyte-based biomimetic NPs	Combination of phospholipids and membrane proteins from leukocytes, followed by incubation with specific antibodies in batch or microfluidic processes	Anti-inflammatory therapy	[67,139]
Aprismatic, enamel-like, nanostructured HAP layers	HAP mineralization from CaCl_2_·2H_2_O and KH_2_PO_4_ in the presence of synthetic peptide solution	Development of enamel-like biomaterials	[41]
MM-camouflaged ROS ^6^-responsive biomimetic NPs	Camouflaging of ROS-responsive polymer NPs with MMs extruded through a 400 nm polycarbonate porous membrane	Atherosclerosis therapy	[46]
Lanthanide NPs-Cas9 ^7^ complex coated with hepatoblastoma cell membrane	Synthesis of NaYF_4_:Yb/Tm/Ca@NaYF_4_:Yb/Nd core–shell NPs from LnCl_3_ aqueous solution, followed by Cas9 binding and coating with hepatoblastoma cell membranes	HBV ^8^-targeted therapy	[47]
NPs functionalized with leukocyte cellular membrane	Biodegradable NPs conjugated with (3-aminopropyl)triethoxysilane, followed by incubation with proteolipid solution	Development of drug delivery carriers	[144]
Anisotropic polymeric NPs coated with RBC membranes	Stretching of spherical PLGA NPs immobilized on a PVA ^9^-glycerol film, followed by sonication-assisted coating with ultrasound-derived RBC membranes	Detoxification of systemically administered bacterial toxin	[33]
BN NP-polydopamine-coated glass fiber-epoxy resin nanocomposite	Facile, water-assisted dopamine coating of glass fiber, followed by addition of BN NPs and epoxy resin components	Development of fiber-reinforced plastic composites	[42]
Al_2_TiO_5_–Al_2_O_3_ ceramics with sea urchin and nacre structure elements	Ball-milling of Al_2_O_3_, SiO_2_, MgO, and TiO_2_, followed by vacuum-drying and pressureless-sintering in air atmosphere	Composite ceramics, catalyst carriers, and sound absorbers	[43]
Ag–TiO_2_ NPs	Sonochemical synthesis of NPs using leaf extract of Origanum majorana as a bioreductant and a stabilizing agent	Antibacterial and antioxidant therapy	[40]
Phosphate-terminated polyamidoamine dendrimer	G4 PAMAM ^10^ modification with dimethylhydrogenophosphonate, followed by treatment with bromotrimethylsilane	Bone and teeth restoration	[145]
Porous SiC coated with Ta	Bioactive metal (Ta) chemical vapor deposition on porous SiC scaffolds	Potential material for bone substitutes	[146]
HAP with multi-scale, hierarchically ordered structure	Self-assembly of layered chitosan–maleic acid matrix, followed by monetite mineralization and transformation to HAP	Developing bone substitute materials	[147]
Amelogenin-containing chitosan hydrogel (modified with enamel proteinase)	Mixing of chitosan solution, CaCl_2_, and recombinant full-length porcine amelogenin, followed by stirring overnight (and addition of enamel proteinase)	Enamel repair	[133,134]
Ceramic biomimetic 3-DOM ^11^ foam	Cork powder pyrolysis to carbon, followed by infiltration with precursor salt solution and calcination to form the oxide ceramic	Environmental and energy applications	[148]
Cellulose nanowhiskers in biopolymer matrices	Microcrystalline cellulose sulfuric acid hydrolysis and centrifugation	Scaffolding in tissue engineering	[149]
Genipin-crosslinked chitosan, alginate, and alumina nanocomposite gels	Alumina powder added to chitosan solution, followed by alginate dissolution and genipin (cross-linking agent) addition	3D bioprinting	[65,150]
Ceramic–organic nanocomposite films	Templated supramolecular surfactant self-assembly on a mica surface	Low-temperature thin-film processing	[151]
Nanometer-sized HAP–collagen composite	Incubation of Tris-buffered CaCl_2_ with sharkskin collagen suspension	Orthopedic implants	[62]
PAMAM ^12^-dendrimer-templated HAP crystallization	Enamel immersion in a solution of CaCl_2_, KH_2_PO_4_, and PAMAM dendrimers modified with carboxylic acid groups	Enamel repair	[135,136,137]
HAP–tricalcium phosphate biphasic NPs	Wet-milling of CaHPO_4_ and CaCO_3_ powders, followed by double-sieving and high-temperature calcination	Bone tissue engineering	[152]
HAP NPs obtained using asparagine–serine–serine peptide	Enamel exposure to triplet repeats of asparagine–serine–serine solution, followed by soaking in artificial saliva	Enamel repair	[63]
Erythrocyte-membrane-camouflaged polymeric NPs	RBC hypotonic treatment and extrusion, followed by mixing with PLGA NPs via extrusion through a porous membrane	Targeted drug delivery	[66]
Monocrystalline ZrO_2_ NPs embedded in an amorphous SiO_2_ matrix	Spark-plasma-sintering of ZrO_2_ NPs and amorphous SiO_2_ powder with a molar ratio of 65% ZrO_2_/35% SiO_2_ at 1200 °C	High-strength translucent glass ceramic materials	[153]
Nacre-like composite of silk nanofibrils, HAP, and chitin nanofibrils	Self-assembly of silk nanofibrils, followed by HAP biomineralization, mixing with chitin nanofibril solution, and nacre-like membrane vacuum-assisted deposition	“Grab-and-release” actuators	[154]

^1^ Hydroxyapatite. ^2^ Poly(lactic acid-glycolic acid). ^3^ Macrophage membrane. ^4^ Zeolitic imidazolate framework. ^5^ Red blood cell. ^6^ Reactive oxygen species. ^7^ CRISPR-associated protein 9 (CRISPR is the clustered regularly inter-spaced palindromic repeat gene-editing therapy). ^8^ Hepatitis B virus. ^9^ Polyvinyl alcohol. ^10^ Generation 4 of polyamidoamine dendrimer. ^11^ Three-dimensionally ordered material. ^12^ Poly(amido amine).

## Data Availability

Not applicable.
